# Perspective on Plasma Polymers for Applied Biomaterials Nanoengineering and the Recent Rise of Oxazolines

**DOI:** 10.3390/ma12010191

**Published:** 2019-01-08

**Authors:** Melanie Macgregor, Krasimir Vasilev

**Affiliations:** 1 School of Engineering, University of South Australia, Adelaide, SA 5000, Australia; melanie.macgregor@unisa.edu.au; 2 Future Industries Institute, University of South Australia, Adelaide, SA 5000, Australia

**Keywords:** plasma polymers, oxazoline, biomaterials, medical devices, implants, coatings

## Abstract

Plasma polymers are unconventional organic thin films which only partially share the properties traditionally attributed to polymeric materials. For instance, they do not consist of repeating monomer units but rather present a highly crosslinked structure resembling the chemistry of the precursor used for deposition. Due to the complex nature of the deposition process, plasma polymers have historically been produced with little control over the chemistry of the plasma phase which is still poorly understood. Yet, plasma polymer research is thriving, in par with the commercialisation of innumerable products using this technology, in fields ranging from biomedical to green energy industries. Here, we briefly summarise the principles at the basis of plasma deposition and highlight recent progress made in understanding the unique chemistry and reactivity of these films. We then demonstrate how carefully designed plasma polymer films can serve the purpose of fundamental research and biomedical applications. We finish the review with a focus on a relatively new class of plasma polymers which are derived from oxazoline-based precursors. This type of coating has attracted significant attention recently due to its unique properties.

## 1. Introduction

**Figure 1:**
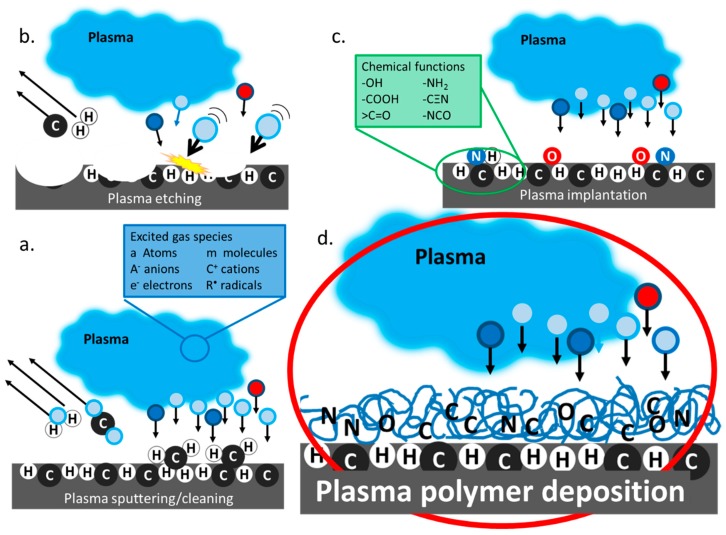
Plasma-assisted surface modification processes (**a**) sputtering (**b**) etching (**c**) implantation and (**d**) polymer deposition.

Plasma in its natural state can be seen as the polar lights and lightning. Far from being rare, this high energy ionised gas phase, referred to as the fourth state of matter, represents more than 99% of the visible universe. Humans artificially induce plasmas using a variety of energy sources including strong magnetic fields, lasers, radiofrequency, electric fields and microwaves. Man-made plasmas are nowadays seen everywhere from commodities such as TV screens, toys and low energy lighting to nuclear reactors and aircraft propulsion turbines. What may be less obvious for some are all industrial uses of plasmas, not as a finished product, but as a manufacturing tool for material surface modification [1]. Plasmas are constituted of highly energetic molecules, molecular fragments, ions, radicals and free electrons. As a result, when plasmas come in contact with solid surfaces, may it be metal, plastic or any other material, they cause important changes to the material surface properties. In this way, it is possible to use plasma-assisted processes to modify a material surface energy, wettability, chemistry and even topography to suits a variety of applications with the added advantage that the properties of the bulk materials are preserved. Plasma-assisted surface modifications encompass a range of techniques which Chu et al. described in quite some detail for the specific case of biomaterials surface modification [2]. Yet, plasma-induced surface treatments processes are used to create novel materials with unique electronic, optical, mechanical and biological properties for many different fields of applications. When the excited species present in the plasma generated from inert (Ar, Ne, He …) or reactive (O_2_, N_2_, NH_3_, CO_2_ …) gases collide with the solid, enough energy may be acquired by the atoms on the surface layer of the solid for them to detach from the surface. When the physical degradation of the bulk material is limited to the outermost layer, the plasma process is called sputtering, Figure 1a [3]. This is the process used in plasma cleaners to remove impurities from contaminated surfaces. If further deeper loss of the exposed material occurs, however, the process is referred to as plasma etching, Figure 1b. Plasma etching is a process in which the adsorption of energetic species is followed by product formation, prior to product desorption [4]. Combination processes have also been reported where anisotropic chemical etching is accelerated by ion sputtering [5]. In biomaterials engineering, plasma is generally used for surface cleaning and sterilisation but at its extreme, plasma etching can also be used for surface roughening and nanopatterning, and even generating novel nanostructures [6,7,8,9]. It is also possible for excited species present in the plasma phase to directly react with the substrate and induce surface modification including the grafting of new chemical functions, such as amine or hydroxyl groups [10]. This process is often referred to as plasma ion implantation. The downside of modifying a surface via plasma sputtering or implantation is that the modification is short lived because reorientation of the surface functional group occurs overtime [11,12,13,14]. Surfaces with lasting properties that completely differ from those of the bulk materials can be generated via plasma polymer deposition. It differs from the other plasma-assisted surface modification in the fact that a thin organic coating is formed over the surface of the original material (Figure 1d). Figure 1 provides an simplified visual representation of the main class of plasma surface modification processes, in which the effect of plasma-wall interaction and particle distribution in the sheath region have not been represented. Important phenomena occurring in this region are still under fervent investigation [15,16]. The figure also does not represent the influence of reactor geometry on the plasma deposition process. The interested reader can refer to an article by Whittle et al. where an international consortium compares coatings produced in 14 different plasma reactors [17].

In this review, we highlight the versatile nature of organic thin films deposited from plasma. We then provide specific examples where carefully designed plasma polymers are used to generate model substrates with controlled surface properties to elucidate fundamental research questions from nanowetting mechanisms to protein adsorption. We illustrate the most recent advances in plasma polymer applications to novel technologies ranging from the development of prosthetic materials, diagnostic devices and even hydrocarbon recovery technologies. Finally, we discuss a new class of plasma polymers that are based on oxazoline precursors pioneered recently by our team. We should stress that this review is not intended to cite all papers published in this exciting field. It is rather intended to summarise recent advances while referring the reader to reviews published by others where a particular aspect of the field is described.

## 2. Plasma Polymers 

Plasma-assisted deposition is a coating technique used to form thin polymer-like films on surfaces. Plasma-enhanced chemical vapour deposition (PECVD) is one of the most common plasma polymerization techniques. It uses plasmas of volatile organic precursors to create polymeric films at low or atmospheric pressure [18]. While plasma polymerization can be performed by a variety of others means such as magnetron sputtering, liquid-assisted deposition, plasma-assisted thermal evaporation, etc., the focus is here given to low pressure PECVD. One intrinsic advantage of PECVD is that it is a dry technique which only uses a minimal amount of precursors and does not produce liquid organic waste. As such, the method is cost effective and environmentally friendly [19,20,21]. The great advantage of plasma polymer deposition compared to conventional techniques for thin film deposition is the capacity to deposit the same surface chemistry with the same conditions on practically any type of substrate material [22,23,24,25]. This is because after the initial deposition of a few angstroms of material, the film growth becomes substrate independent [22,23]. In contrast, techniques for surface modification such as layer-by-layer (L-b-L) and Self Assembled Monolayers (SAMs) require substrates with highly specific properties, which narrows opportunities for applications [26].

The films generated are commonly referred to as plasma polymers although they do not formally classify as polymers because they do not consist of repeating monomer units. Instead, they are formed of a variety of precursor fragments and recombination products, and are generally highly crosslinked. Historically, such films have been produced with little control over the chemistry of the plasma phase. Despite the use of advanced techniques directly in the plasma phase such as Mass Spectroscopy, Langmuir probes and Optical Emission Spectroscopy, the mechanisms of plasma polymerization remain poorly understood [27,28,29,30]. However, advances in surface characterization techniques and the pull for applications have fueled much progress in this area, and it is now possible to control deposition conditions in many ways so that chemistry and functionality of the resulting coating can be finely tailored to suit specific applications ranging from wearable electronics [31] and solar cells [32] all the way to water treatment [33]. It is also possible to deposit plasma polymers onto micro and nanomaterials as well as powders. This approach has been used to create adsorbents for water treatment purposes. Silica or magnetic nanoparticles can be coated with a hydrophobic plasma polymer layer to remove hydrocarbon residue from waste water [33,34]. Thiophene-coated particles are able to isolate heavy metals [35], and allylamine-coated powders successfully remove dyes from waters which is relevant for the leather, textile, paper, pharmaceuticals, paper and food industries [36]. Plasma polymers coatedmagnetic nanoparticles were even recently used to remove haze proteins from wines [37,38].

The first degree of freedom when designing a plasma polymer is the monomer choice amongst a wealth of precursors [39]. Plasma polymers can be deposited from practically any compound volatile enough to be introduced into the reaction chamber. Table 1 provide examples of organic precursors commonly used to produce thin polymeric films from their plasma phase. This list includes monomers like ethanol which are difficult to polymerise via conventional means but can be deposited into a polymeric film using plasma processes [29,40]. It also includes examples of precursors containing sulfur and fluoro heteroatoms which can provide very interesting reactivity and wetting properties. Another interesting example is that of oxazolines. Following classic organic chemistry routes, oxaolines are polymerized via ring opening polymerization which results in a linear polymer with amide repeating units. Several works have demonstrated that the plasma deposition of oxazolines generates surface chemistries that are not achievable via conventional means, including the formation of isocyanate and nitrile groups but also the retention of the oxazoline ring itself. The presence of intact oxazoline rings provides unique opportunities to conduct binding reactions of biomolecules, nanoparticles and various ligands that carry carboxyl acid groups in their structures [41,42]. Other groups have investigated the plasma deposition of other ring-containing monomers with heteroatoms including pyrrole [43], furfuryl [44], thiophene [45], aniline [46] and even essential oils [47,48,49]. Plasma polymers prepared from non-synthetic monomers are a particularly hot topic because they combine desirable optical and physical properties with biocompatibility and environmental sustainability [50,51]. However, as the complexity of the monomer increases, so does the importance of carefully tuning the plasma deposition condition to tailor the amount of functionality retention to suit any specific application [52,53] and ensure that film reactivity can be maintained for relevant aging time [54].

## 3. Plasma Polymers in Biomaterial Research

Plasma polymers are a coating of choice for biomedical applications. The topic has been extensively reviewed elsewhere [76,77]. Plasma polymer deposition enables the generation of surfaces where the entire spectrum of surface properties including chemistry, wettability, stiffness and nanotopography can be precisely tailored. The technique is rapid and reproducible; thus, it is possible to create large quantities of model surfaces with well-controlled properties for the investigation of many complex processes, ranging from protein binding, to immune response through to the fundamentals of nano-wetting. They are also underpinning new biosensing platforms [78] and novel cell guidance surfaces. We used a capacitively coupled parallel plate plasma chamber [22] to generate films from a variety of organic precursors including allylamine, octadiene, aldehyde, ethanol, acrylic acid or even perfluorooctane. The resulting coatings present distinctive chemical functions, namely, amines, hydroxyls, carboxylic acids, ketones, etc. These plasma polymers have been used as a utility to investigate the systematic effect of surface chemistry on many biological processes including the biofunctionality of surface-adsorbed proteins [40], the differentiation of embryonic [79], kidney [80], dental pulp [81], mesenchymal [82] and human adipose-derived stem cells [83,84] as well as the deposition of collagen by primary human dermal fibroblasts [85]. Using the reactivity of the chemical groups created, it is also possible to bind ligands and even proteins to create diagnostic tools [40,86,87,88,89]. Fine-tuned chemical functionality facilitated by plasma polymers also allowed binding to surfaces of gold and silver nanoparticles to generate model surface nanotopography to study biological phenomena or solve environmental challenges [25,90,91,92,93], and nanoengineered surfaces with controlled nanofeatures size and density [94]. In this manner, uniform and gradient nanotopography can be generated using electrostatic binding of gold or silver nanoparticles capped with mercapto succinic acid to allylamine plasma polymers or covalent binding to oxazoline-based coatings [95]. A thin layer of plasma polymer can then be deposited on top of the surface nanotopography to control the outermost surface chemistry while preserving nanotopography. This is a unique approach that can be achieved only by plasma polymerization or iCVD. An important application of this approach was to derive understanding of the influence of surface nanotopography and chemistry on inflammatory responses [96,97]. Such knowledge is vital for the utilization of plasma polymers in implantable devices [98,99]. Thanks to the wide range of intrinsic surface chemistry that can be achieved, the films created in this way span a uniquely wide range of wettability, generating water contact angles from 20° to 120° on smooth surfaces. When plasma polymers are combined with nanotexturing, remarkable wetting states such as superhydrophobicity and superhydrophilicity can be achieved [100,101,102]. We nanoengineered model gradient substrates with intrinsic wettability ranging from hydrophilic to hydrophobic to investigate the mechanisms governing wetting at the nanoscale. While classical theories could not account correctly for the water contact angles measured on nanorough surfaces, we were able to develop an empirical model that effectively captures the experimental data. The model, which is now known as the Vasilev-Ramiasa equation [103], further enables us to predict the water contact angle on the nanorough surfaces, using as the only known parameter the number density and size of the spherical nanofeatures and the contact angle on the smooth substrate itself. A range of other biomedical applications have also been facilitated by plasma polymers including in drug delivery allowing to achieve controlled release rate of synthetic therapeutics or biomolecules [104,105,106].

Another particularly useful application of plasma polymers is in antibacterial technologies [107,108,109]. Coatings able to protect the surface of medical devices from bacterial adhesion and biofilm formation are used to minimize the risk of infection, and especially hospital acquired infections which are one of this century’s biggest health concerns. Infections are a well-studied subject and it is now well-known that the attachment of individual planktonic bacterial cells to the device surface is just the first step, followed by colonization and infection. It is also well understood that once a biofilm is formed, it protects the bacterial cells from the immune system and enormously (up to 1000 times) increases the dose of antibiotics required to clear the infection. This overuse of antibiotics leads, on the one hand, to development of antibiotic resistance by bacteria and, on the other hand, causes systemic toxicity to organs such as the kidneys and liver. For these reasons, the purpose of antibacterial coatings is to disturb the initial stage of bacterial adhesion [110,111]. This can be achieved through one of four distinct mechanisms of action: contact killing, bacterial repellence, killing in solution or stimuli responsive killing [109].

Plasma polymers have been used to generate contact killing surfaces on several occasions [112,113]. We conducted a study in which quaternary ammonium compounds were covalently immobilized to allylamine plasma polymers and found that surface concentration of NR_4_^+^ groups equivalent to 5 At% nitrogen and surface potential of +120 mV was necessary to damage bacteria. In another study of surface-bacteria interaction, plasma processes were used to generate silicon nanospikes which we coated with different plasma polymer thin films to tailor the substrate chemistry and wettability [114,115]. We found that for hydrophilic substrates, the presence of nanospikes resulted in bacterial death on contact. Arguably, the most efficient way to limit bacterial colonization is to use coatings that release antimicrobial agents either passively or in response to external stimuli. Our group has explored numerous avenues to produce such platforms using plasma polymers, including the ‘sandwiching’ of antibiotics between two plasma polymer layers [106,116], a sacrificial nano-templating approach to create antibiotic reservoirs whose release could be controlled by a plasma polymer overcoating [105] or encapsulation of antibiotic particulates within plasma polymers. As silver is known as a potent antibacterial agent, much work has also been done revolving around the use of silver nanoparticles for the release of silver ions [91,92,117,118,119,120,121]. The application of silver in antibacterial technology is reviewed in much detail elsewhere [122]. Nitric oxide releasing as well as new TEMPO-based coatings capable of delaying biofilm growth but stimulating mammalian cells growth has also been reported [123,124,125]. The potential of oxazoline-based plasma polymer coatings that we recently developed in antibacterial technologies is discussed later in this review.

## 4. Plasma Deposited Polyoxazolines—The Importance of Deposition Conditions

For most plasma polymers, chemistry, functionality retention and reactivity are highly dependent on the plasma deposition parameters. For instance, it is well accepted that high powers and low flow rates lead to greater monomer fragmentation and crosslinking. The films deposited using these conditions are typically more stable than those generated at low powers and high flow rates [106,126]. In contrast, low flow rate and high powers allow for the retention of the original precursor functionality which results in films that are relatively more reactive. Carefully balancing the precursor flow rate and power is essential to induce film growth, rather than substrate etching [20]. Several studies investigating the growth and adhesion properties of plasma-deposited polymer films have shown that excellent adhesion can be achieved on a variety of substrates [24]. Furthermore, in the film growth regime, increasing deposition time increases film thickness and it was proven that beyond 5 nm in thickness, the nature of the underlying materials does not affect the properties of the polymer films [22,23,24]. All in all, almost every time a new precursor is used for plasma deposition, optimization is required, even more so when the precursor contains 1 or more heteroatoms.

### 4.1. PPOx Physico-Chemical Characterization

In the case of plasma-deposited oxazoline films, careful optimization of the deposition parameters is paramount [127].

**Figure 2:**
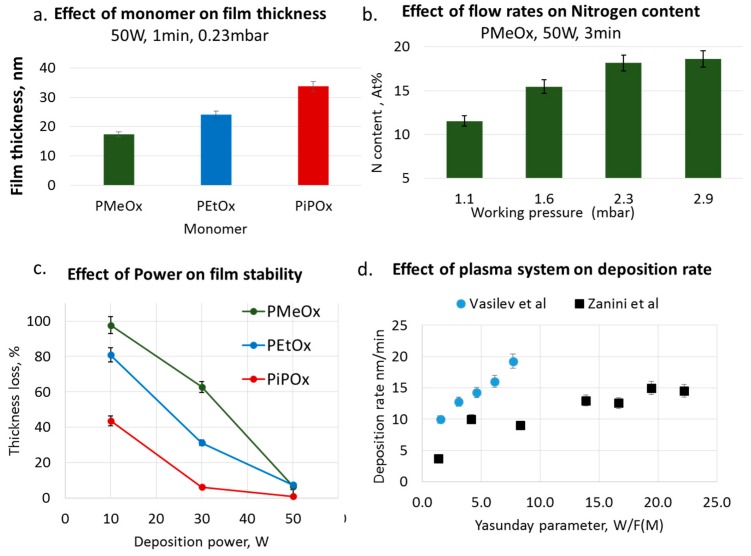
PPOx deposition conditions: (**a**) Effect of monomer chemistry on film thickness, (**b**) precursor flow rates on nitrogen content, (**c**) of plasma ignition powers on films stability for MeOx, EtOx and PiPOx. (**d**) PPEtOX Plasma deposition rate as a function of the Yasunda parameter is defined as the ratio between plasma power and monomer flow rate, in two different plasma reactors.

Our research was motivated by the fact that the conventional polyoxazoline polymer (POx) displayed interesting biomaterial properties such as good biocompatibility, low cytotoxicity and excellent stealth properties with stability rates surpassing those of PEG-modified surfaces. The development of the first generation of plasma deposited x oxazolines conducted by our group revealed that, through the plasma process, we could not only produce films with comparable properties but also films with added reactivity. These results illustrate the importance of carefully controlling deposition conditions to tailor the plasma-coating properties [42]. Our group pioneered the field of plasma-deposited oxazolines (PPOx) using various oxazoline precursors (methy, ethyl and isopropylene) and a range of plasma ignition powers, monomer flow rates and deposition times (Figure 2 and Table 2). The films were deposited in a custom-made inductively coupled glass chamber plasma reactor described in detail elsewhere, in which the brass electrodes are set 10 cm apart [128]. The plasma ignition power was varied from 10 to 50 W. The incoming precursor flow rate, referred to as the chamber working pressure, was adjusted between 0.10 and 0.30 mbar. The deposition time ranged from 1 to 7 min. The plasma phase was characterised using in-situ Mass Spectrometry, and PPOx films were characterised via Ellipsometry, Water Contact Angle measurement, X-ray photoelectron, Fourier Transform Infrared Spectroscopy and Time of Flight Secondary Ion Mass Spectrometry. By tuning the deposition conditions, we could produce plasma-deposited polyoxazoline (PPOx) thin films stable under various pH and salt conditions that were biocompatible and chemically reactive [42]. Our results were soon confirmed by other groups who specifically studied films formed from 2-ethyl-2-oxazoline, Figure 2d [129,130].

**Figure 3:**
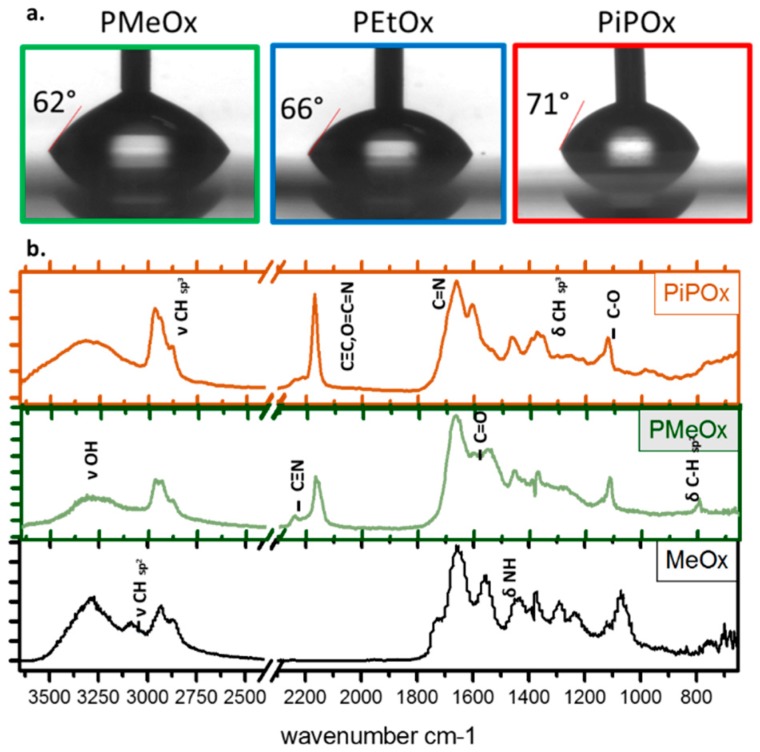
(**a**) Advancing water contact angle of water in air on plasma-deposited Methyl, Ethyl, and isopropenyl oxazoline deposited at 50 W and 2.3 mbar. (**b**) FTIR spectra of isopropenyl, and methyl oxazoline deposited under the same conditions as well as a pristine methyl oxazoline precursor.

The films produced by Bhatt et al. have comparable chemistry, but the use of a lower plasma ignition power resulted in significant film loss after water exposure. Our results, shown in Figure 2c, also show that film loss occurred after 1 h immersion in water for the films deposited at the lowest power (10 W). Zanini et al. also used low pressure depositions condition and investigated a wider range of ignition powers, including some comparable to the ones used in our experiment (3.9 sccm, and powers from 5 to 80 W). Table 2 summarizes side by side the reactor specifications and deposition conditions used by these two groups. Comparing our results and those of Zanini et al. for 2-ethyl-2-oxazoline films deposited in two different plasma reactors and using different precursor flow rates, Figure 2d, we found film deposition rates of the same order of magnitude and comparable film wettability, in the range of approximately 60 to 70 depending on the nature of the precursor and deposition conditions, Figure 3a. It is worth noting, however, that the relationship between the film deposition rate and the power input (W/F) is complex and will be influenced by the plasma reactor geometry (e.g., deposition area and electron energy distribution function, ion density) despite the fact that the deposition conditions described in Zanini et al. appear comparable to those used in our study [27,52,131,132]. As such, it is only meaningful here to compare the macroscopic attribute of the films as their intrinsic chemical nature may be quite different. 

### 4.2. PPOx Unique Reactivity

**Figure 4:**
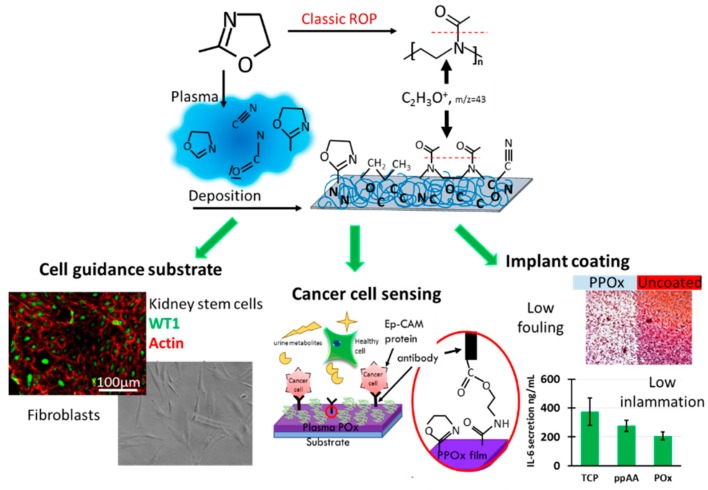
Schematic illustrating the PPox deposition process (**top**) and its applications (**bottom**) for cell guidance surfaces, diagnostic devices and low fouling properties. The left images illustrate the biocompatible nature of PPOx coating on which multiple cell types including human dermal fibroblast and kidney stem cells proliferate as successfully as on tissue culture plate. The middle schematic illustrates the reaction occurring between PPOx and biomolecule with COOH function and how this biofunctionalisation is used for the selective capture of cancer cells. The right-hand side shows the inhibited proliferation of biofilm on PPOx substrates as well as the decrease in pro-inflammatory cytokine IL6 secretion, which together makes PPOx a suitable candidate for implant coatings.

Most importantly, these studies from different research groups demonstrated that under all deposition conditions, the films displayed a rich film chemistry of amine, amide, carbonyl, carboxyl and nitrile functions, as well as intact oxazoline rings [42,130]. Figure 3b presents a typical Fourier Transform Infrared Spectroscopy (FTIR) spectrum for oxazoline films deposited from methyl and isopropenyl oxazoline as well as the intact methyl precursor for direct comparison. However, the relative concentration of the surface chemical group, as well as the film wettability and stability, varies with plasma power and the precursor flow rate, as described in details previously [42,127]. Advanced in situ Mass Spectroscopy of the plasma phase correlated with Tof SIMS principal component analysis provided important insights into the physicochemical event occurring in the plasma itself and post deposition [30]. This study revealed that amide groups present in the plasma deposited oxazolines are the result of post deposition reactions in the films while functional groups such as nitrile, isocyanates and oxazoline ring are formed in the plasma process itself and are better retained in the final films when gentle deposition conditions are used. A schematic representing the resulting chemical group present in the PPOx film is shown in Figure 4. Zanini et al. used NMR to estimate the amount of oxazoline ring retention as well as the degree of linear open ring structure in PPOx surfaces. In PEtOX film deposited at 15 W, they achieved 20% ring retention, while the remaining of the surface chemistry resulted from more complex plasma fragmentation and recombination processes, in good agreement with our spectroscopic investigations. The distinctive oxazoline ring functionality of PPOx is a clear advantage over conventional POx because the ring can form covalent bonds with carboxylic acid groups present in biomolecules and in other ligands [42].

This unique reactivity of PPOx with –COOH functionalities, which was also confirmed by Zanini et al., has been used to create surfaces with controlled nanotopography by covalent binding of COOH– functionalised nanoparticles [94]. In a recent study, we created in this way a range of substrates with different nanoparticle sizes and densities to interrogate the effect of nanotopography on protein binding using PPOx as the overlayer [133]. It is well accepted that cell adhesion to biomaterials relies on the initial formation of an adsorbed protein layer [134]. As cells come into contact with the protein film, integrin receptors on the cell membranes recognize favorable binding sites which initiates the adhesion process. It is therefore essential for biomaterials to promote the adsorption of bioactive proteins. Oxygen-rich and nitrogen-rich plasma-derived surfaces have been particularly studied for their application as biomaterials [135]. Oxygen-rich surface chemical functions are known to promote cellular attachment because they contain polar groups such as hydroxyls [136], carboxyls [137,138] and carbonyls [57], which enter ionic interactions with cell adhesion-mediating molecules [139]. Amine-rich plasma polymers, prepared from allylamine [61,62], ethylendiamine [67], propylamine [68], butlyamine [69] and heptylamine [70], also are coatings of choice for biomedical applications [140]. They are positively charged in cell culture conditions and facilitate the electrostatic adsorption of negatively charged proteins. This protein binding ability is thought to confer the amine rich surfaces their biocompatiblity. Plasma-deposited polyoxazolines are both oxygen and nitrogen rich. Surface chemistry analysis indicated that oxygen and nitrogen are, in a PPOx film, engaged in many hydrophilic, H-donor groups which may explain their good ability to support mammalian cell growth, as evidenced in our studies of oxazolines as cell guidance surfaces [41]. We found that mammalian cells such as kidney stem cells and fibroblast do adhere and proliferate on POx coated surfaces just as well as on control tissue culture plate, Figure 4, left. Our work on protein adsorption to nanorough PPOx-coated surfaces demonstrated that the amount of protein bound to the surface is determined by nanotopography-induced geometric effects and surface wettability rather than overall surface area. Protein adsorption was hindered on a densely packed array of 16 and 38 nm gold nanoparticles overcoated with PPOx compared to smooth PPOx substrates, while it increased for 68 nm nanoparticles [94,141].

More recently we conducted a one-year aging study of PPOx films which demonstrated that the reactivity of the films towards COOH-functionalised gold nanoparticles [142] was retained above 90% when the films were kept in dark, vacuum-sealed containers [143]. This extended shelf live is a significant asset for future commercial applications of PPOx. From our detailed investigations, we compiled a matrix of the physico-chemical properties of PPOx as a function of the plasma deposition conditions that we used to design PPOx films tailored for cell guidance surfaces, biosensors and low fouling substrates as explained below, and depicted in Figure 4.

### 4.3. PPOx in Novel Technology

An enormous advantage of plasma polymers for the industrialization of novel technologies is that they can be deposited on any type of substrate from the four classes of materials (i.e., metals, ceramics, polymers and composites) including those featuring complex shapes and topography. The capacity to preserve valuable bulk properties but to alter the properties at the surface contributes substantial added value to numerous products in fields ranging from medicine to membrane filtration and electronics.

Oxazoline-derived plasma polymer coatings are a promising candidate in antibacterial technologies. Our research demonstrated that, using appropriate deposition conditions, bacteria may attach in small numbers to the PPOx but would not proliferate to form biofilms, Figure 4, right [144]. While the mechanisms underlying these interesting properties are still under investigation, the simplicity of the method has already attracted industrial interest as it provides excellent opportunities for developing medical device-coating technologies. This property of PPOx films is interesting for their use as low fouling coatings for implantable devices. For this reason, we also investigated the response of immune cells to PPOx films. In this investigation, cytokine secretion from bone marrow-derived primary macrophages (BMDM) was measured in vitro. BMDM were selected as model immune cells because their function is to mediate early innate immune inflammatory responses [41,141]. Compared to other nitrogen-rich plasma polymer and tissue culture plates, a marked reduction in the expression of TFN-α and IL-6 cytokine was observed on the PPOx substrates, Figure 4, right. Together, these results indicate that PPOx films could benefit several device types such as prosthetics, catheters, or even wound dressings. 

Bladder cancer diagnostic devices: The PPOx capacity to spontaneously form covalent bonds with the carboxylic acid groups present in biomolecules was used to generate immunofunctionalised surfaces for the selective capture of cancer cells from urine [145]. Anti-epithelial cell adhesion molecule antibodies were covalently bound to PPOx substrates. Using PPOx to immobilise antibodies for diagnostic purposes is very useful because the strong covalent bond between the substrate and the sensing biomolecule is not disrupted by physiological variations in the composition, pH, and ionic strength of real body fluid such as urine [146]. The biosensors developed in this way were able to detect cancer cells in model urine and also in real patient urine samples. The outcomes of this work are currently being tested in the clinical setting.

## 5. Conclusions and Outlook

Collectively, this review summarizes the principles at the basis of plasma deposition and highlights recent progress made in understanding the unique chemistry and reactivity of coatings deposited by this method [147]. We then demonstrate how carefully designed plasma polymer films can serve the purpose of fundamental research and biomedical applications. We placed emphasis on relatively recently developed oxazoline precursor-derived plasma polymer coatings which have been demonstrated to offer a number of intriguing and very valuable properties. Some of these properties are unique such as the retention of a population of intact oxazoline rings on the surface of the coatings which opens new opportunities in bio-sensing and medical diagnostics. Other valuable properties such as elimination of biofilm formation and excellent biocompatibility make these coatings good candidates for surface modification of implantable medical devices, tissue engineering constricts, cell and drug delivery vehicles, and many others. However, this is just the beginning of a new era. With more researchers and companies adopting the technology, many new interesting properties will be discovered and new opportunities for commercial applications will be identified. 

## Figures and Tables

**Table 1:** Examples of common organic precursors used to prepare plasma-deposited film with different surface chemistry.

Precursor	Chemical Formula	Surface Functionality	Ref.
Acrylic acid	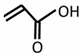	carboxyl	[55,56,57]
Allylalcohol	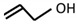	hydroxyl	[58,59,60]
Ethanlol	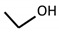		
Allylamine	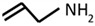	Amine, amide	[61,62,63,64]
Allylglycidyl ether	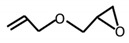	Epoxy	[65,66]
Glycidyl methacrylate	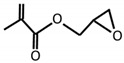		
Alkyloxazoline	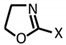	Oxazoline, amine, amide	[41,42]
Ethylene diamine	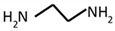	Amine, amide	[67,68,69,70]
Alkylamine	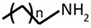		
Propanal	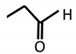	Aldehyde	[40,71,72,73]
1,7-octadiene	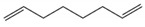	Alkyl	[56]
perfluoroocatane	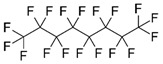	Fluoro	[34]
Propanethiol	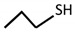	Thiol	[74,75]

**Table 2:** Plasma reactor specification and depositions condition ranges for the PPOx deposition studies conducted by Zanini et al. and Vasilev et al.

Reactor and Deposition Parameters	Zanini et al.	Vasilev et al.
Vacuum chamber	Stainless steel	Glass
Chamber Diameter, cm	30	15
Electrode	Stainless steel	Brass
Electrode Diameter, cm	15	10
Separation Distance, cm	4	10
Monomer input	Showerhead, 2 mm pinholes	Single inlet, 5 mm
Radio frequency, MHz	13.56	13.56
Base pressure, Pa	10^−3^	10^−1^
Working pressure, Pa	6	1–3
Power range, W	4–80	10–50
Deposition time, min	10–30	1–7
